# The correlation between morphological parameters and the incidence of de novo chromosomal abnormalities in 3238 biopsied blastocysts

**DOI:** 10.1007/s10815-023-02780-5

**Published:** 2023-04-14

**Authors:** Jiangman Gao, Nan Wei, Xiaohui Zhu, Rong Li, Liying Yan, Jie Qiao

**Affiliations:** 1 Center for Reproductive Medicine, Department of Obstetrics and Gynecology, Peking University Third Hospital, Beijing, 100191 China; 2 National Clinical Research Center for Obstetrics and Gynecology, (Peking University Third Hospital), Beijing, 100191 China; 3 Key Laboratory of Assisted Reproduction (Peking University), Ministry of Education, Beijing, 100191 China; 4 Beijing Key Laboratory of Reproductive Endocrinology and Assisted Reproductive Technology (Peking University Third Hospital), Beijing, 100191 China

**Keywords:** Embryonic developmental competence, Blastocyst morphology, Whole chromosomal aneuploidy, Segmental chromosomal aneuploidy, Mosaicism, PGT-M

## Abstract

**Purpose:**

The aim of this study was to determine the relationship between morphological parameters and the incidence of de novo chromosomal abnormalities.

**Methods:**

This was a retrospective cohort study of 652 patients who underwent 921 cycles with 3238 blastocysts biopsied. The embryo grades were evaluated according to Gardner and Schoolcraft’s system. The incidence of euploidy, whole chromosomal aneuploidy (W-aneuploidy), segmental chromosomal aneuploidy (S-aneuploidy), and mosaicism in trophectoderm (TE) cell biopsies was analyzed.

**Results:**

The euploidy decreased significantly with maternal age and was positively correlated biopsy day and morphological parameters. The W-aneuploidy increased significantly with maternal age and was negatively correlated biopsy day and morphological parameters. Parental age, TE biopsy day, and morphological parameters were not associated with S-aneuploidy and mosaicism, except that TE grade C blastocysts had significantly higher mosaicism than TE grade A blastocysts. Subanalysis in different female age groups showed that euploidy and W-aneuploidy had a significant correlation with TE biopsy day among women aged ≤ 30 y and 31–35 y, with expansion degree among women aged ≥ 36 y, with ICM grade among women aged ≥ 31 y, and with TE grade among all female age ranges.

**Conclusion:**

Female age, embryo developmental speed and blastocyst morphological parameters are associated with euploidy and whole chromosomal aneuploidy. The predictive value of these factors varies across female age groups. Parental age, embryo developmental speed, expansion degree, and ICM grade are not associated with the incidence of segmental aneuploidy or mosaicism, but TE grade seemingly has a weak correlation with segmental aneuploidy and mosaicism in embryos.

## Introduction

Blastocyst culture and transfer have been increasingly used in assisted reproductive technology (ART). This approach has several theoretical advantages, such as the opportunity to potentially select the most viable embryo(s) for transfer, better temporal synchronization between the embryo and endometrium at the time of embryo transfer, higher implantation and live-birth rates than those with cleavage-stage embryo transfer, and potential decreases in multiple pregnancy rates and the risks of maternal and fetal morbidity by single blastocyst transfer[1–3].

Embryo quality is traditionally evaluated based on morphological characteristics. The blastocyst morphology grade system introduced by Gardner and Schoolcraft is the most accepted and widely used to select blastocysts in IVF. The system is based on the degree of blastocyst expansion and the morphology of the inner cell mass (ICM) and trophectoderm (TE) cells [4, 5]. Some studies have reported that blastocyst morphology parameters are significantly associated with the implantation rate, clinical pregnancy rate, ongoing pregnancy rate, and live birth rate[6–10]. Additionally, the blastocyst development rate affects clinical outcomes, evidenced by the fact that day-5 blastocysts have significantly higher implantation, clinical pregnancy, and live birth rates than day-6 blastocysts[11, 12].

Chromosomal abnormalities in preimplantation embryos are common[13]. In many cases, the presence of aneuploidy is proposed to be the underlying cause of embryonic arrest, implantation failure, and spontaneous pregnancy loss in natural pregnancy and ART. Approximately 50–70% of all miscarriages are due to aneuploidy, and eliminating aneuploid embryo transfer could significantly reduce the rate of miscarriage after IVF[14]. Selection of single euploid blastocysts for transfer is more likely to result in implantation and development into a healthy pregnancy, avoiding multiple pregnancies and related complications[15, 16]. Previous studies reporting cytogenetic data from oocytes and embryos focused primarily on the incidence of whole chromosome abnormalities. However, segmental aneuploidy, losses or gains of chromosomal fragments, also occur at appreciable frequencies in preimplantation embryos[17, 18]. The frequency of de novo segmental aneuploidies in blastocysts detected by TE biopsies is approximately 5–12%[19–21]. Mosaicism, a mixture of cells with different karyotypes, is another abnormality found in the preimplantation embryo[22, 23]. Although the correlation of blastocyst morphology parameters with ploidy and clinical outcomes has been previously reported, to our best knowledge, no work has been reported to identify the relationships of blastocyst development, expansion, ICM grade, and TE grade with the incidence of whole chromosomal aneuploidies, segmental chromosomal aneuploidies, and mosaicism detected in TE biopsies at the same time.

Preimplantation genetic testing for aneuploidy (PGT-A) is an embryo selection technique used for women of advanced maternal age, those with recurrent pregnancy loss, and patients with recurrent IVF failure or an abnormal chromosome karyogram[24]. The application of PGT to monogenic disorders (PGT-M) represents several important advances, including parallel analysis of mutation-linked markers and prevention and detection of DNA contamination, which helps avoid misdiagnosis and improve accuracy[22]. Monogenic disorders are caused by pathogenic variations in a single gene, which theoretically would not affect the frequency of aneuploidy in embryos in most cases.

To further investigate the association between blastocyst chromosomal abnormalities and morphological characteristics, we conducted a retrospective cohort study with a large sample size in a single center to analyze the correlations of maternal and paternal age, developmental competence of embryos (biopsy days), blastocyst expansion degree, ICM grade, and TE grade with euploidy and the incidences of whole chromosomal aneuploidy, segmental chromosomal aneuploidy, and mosaicism from TE biopsy at the blastocyst stage.

## Materials and methods

### Patients and study design

The data were collected from May 2015 to July 2022 at the Center for Reproductive Medicine. We enrolled 652 couples carrying monogenic diseases who sought fertility treatment for PGT-M in this retrospective cohort study. The average female age was 31.8 years (range 22–43 years). A total of 921 cycles were performed. Patients with chromosome translocation or Y-chromosome microdeletion were excluded. Cycles, in which no blastocysts were obtained, or blastocyst grading was not recorded, were also excluded.

### Clinical protocol

Assisted reproductive methodologies, including controlled ovarian hyperstimulation, oocyte maturation trigger, and oocyte retrieval, were carried out according to standard protocols used at the Center for Reproductive Medicine. Briefly, controlled ovarian stimulation was applied using the GnRH antagonist protocol, oocyte maturation was triggered through hCG administration, and ultrasound-guided ovum retrieval was performed approximately 36 h after hCG injection. Mature oocytes at the MII stage were fertilized by intracytoplasmic sperm injection (ICSI) on the day of egg retrieval, and embryos were cultured for 5–7 days in vitro.

### Blastocyst morphological classification and trophectoderm biopsies

Morphological assessments were conducted on the day of TE biopsy (day 5, day 6, or day 7). Blastocysts were graded according to three separate quality scores: the degree of expansion (1–6), the grade of ICM (A, B, C), and the grade of TE (A, B, C) according to Gardner and Schoolcraft’s system[4].

All blastocysts for PGT were artificially hatched on day 3 after fertilization. TE biopsy was performed by laser-assisted methodologies on embryos with expansion grades 4 or above on days 5, 6, or 7. Four to six TE cells were gently aspirated with a biopsy pipette from the trophectoderm of each blastocyst. The biopsied TE cells were washed in sterile phosphate-buffered saline (PBS) solution and then kept in a 0.2-mL polymerase chain reaction (PCR) tube with 5 μl of lysis buffer. After TE biopsy, the blastocysts were cryopreserved by vitrification.

### Preimplantation genetic testing and definition

The whole genome of the biopsied TE cells was amplified with a multiple annealing and looping-based amplification cycle (MALBAC) Single-Cell WGA Kit (Yikon Genomics Inc., China) and analyzed using next-generation sequencing (NGS) as described previously[25]. Blastocysts analyzed by TE cell biopsy were classified as euploid, aneuploid or mosaic. All aneuploidies were further classified as whole-chromosome aneuploidy (W-aneuploidy) or segmental-chromosome aneuploidy (S-aneuploidy). In the data analysis, blastocysts with uniform single or multiple whole-chromosome aneuploidies were classified as W-aneuploid blastocysts; blastocysts with one or more segmental aneuploidies were classified as S-aneuploid blastocysts, and blastocysts with at least one mosaic chromosome abnormality were classified as mosaicism blastocysts.

### Statistical analysis

All data analyses were performed using IBM SPSS Statistics software version 23.0. The continued data are presented as the mean and standard deviation (SD). The categorical variables are presented as counts (percentages) and were analyzed by a chi-squared test. In the multivariate logistic regression analysis, female age, male age, biopsy day, and blastocyst morphological parameters (expansion degree, ICM grade, and TE grade) were included, and the data are reported as odds ratios (ORs) and 95% confidence intervals (95% CIs). *P* < 0.05 was considered statistically significant.

## Results

### Baseline characteristics and description

A general overview of the study population and embryos is presented in Table 1.

**Table 1:** Characteristics of the study population and embryos

Characteristic	Description
Number of couples, n	652
Number of cycles, n	921
Mean female age, (years, ± SD)	31.8 ± 4.0
Mean male age, (years, ± SD)	33.2 ± 4.7
Number of retrieved oocytes, n	13979
Mature oocytes, n (%)	10969 (78.5%)
Number of cleavage embryos, n (%)	8102 (73.9%)
Blastocysts obtained, n (%)	3238 (40.0%)
Day of biopsy, n (%)	
Day 5	710 (21.9%)
Day 6	2462 (76.0%)
Day 7	66 (2.1%)
Blastocyst expansion, n (%)	
4	83 (2.6%)
5	2523 (77.9%)
6	632 (19.5%)
ICM grade, n (%)	
A	446 (13.8%)
B	2604 (80.4%)
C	188 (5.8%)
TE grade, n (%)	
A	424 (13.1%)
B	1960 (60.5%)
C	854 (26.4%)
Blastocysts successfully analyzed (n, %)	3149 (97.3%)

Continuous variables are expressed as median ± standard deviation (SD) and categorical values are presented as number (percentages). ICM, inner cell mass, TE, trophectoderm

**Fig. 1:**
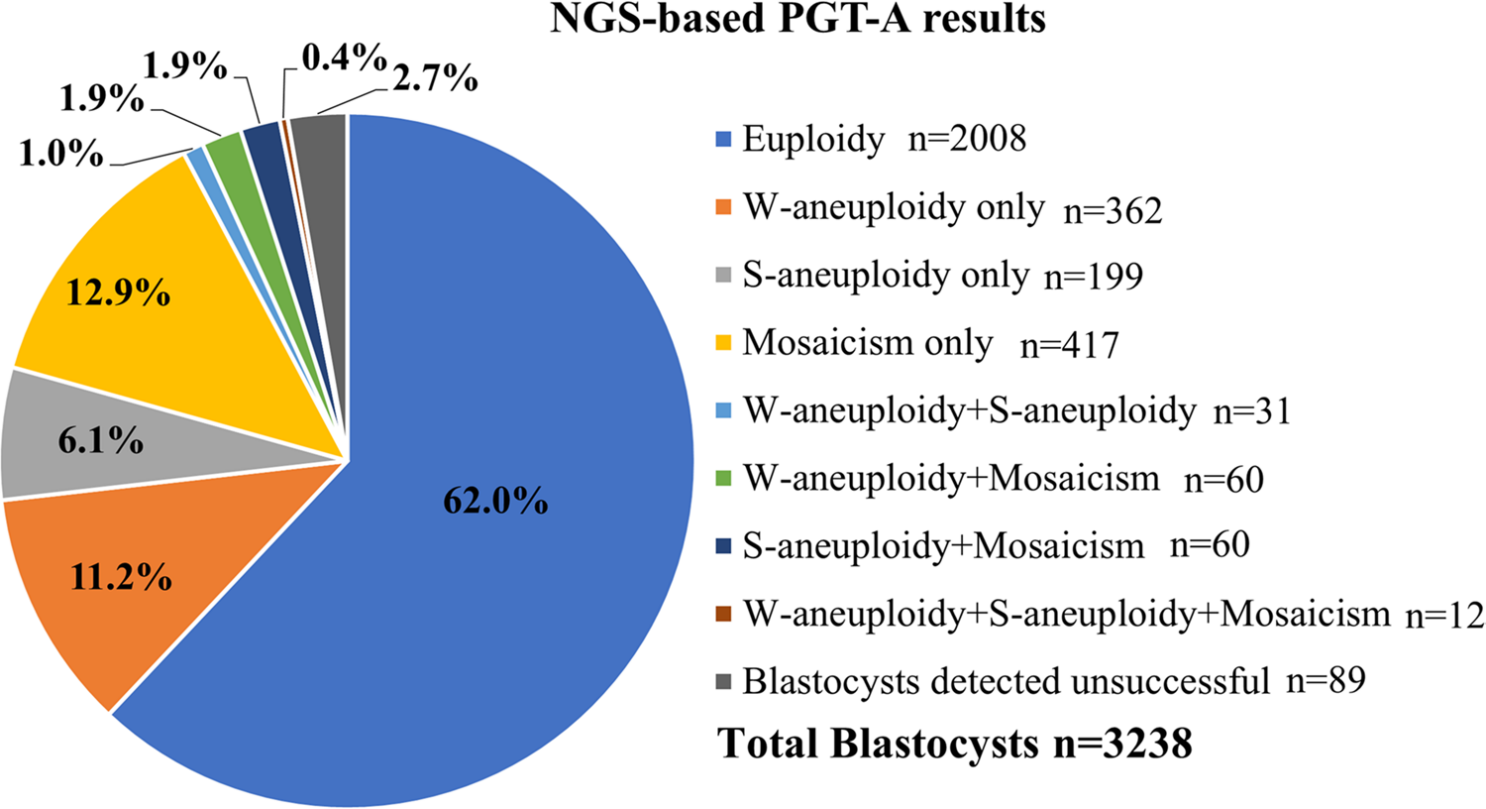
Summary of NGS-based PGT-A results in all biopsied blastocysts

A total of 921 cycles, including 3238 blastocysts from 652 patients, were included in this study. The average age of the female patients was 31.8 ± 4.0 years (range 22–43 years), and the average age of the male patients was 33.2 ± 4.7 years (range 22–56 years). The total number of retrieved oocytes was 13,979, including 10,969 (78.5%) mature (metaphase II, MII) oocytes. The number of cleavage-stage embryos was 8102 (73.9%). A total of 3238 (40.0%) blastocysts were obtained, including 710 (21.9%) blastocysts on day 5, 2462 (76.0%) blastocysts on day 6, and 66 (2.1%) blastocysts on day 7. Among these blastocysts, more than 97% (3149/3238) of NGS-based TE biopsies were successfully analyzed. Figure 1 shows the results of genetic testing for aneuploidies. In all tested blastocysts, 62.0% (2008/3238) of blastocysts were euploid. The percentage of blastocysts with at least one whole aneuploidy was 14.4% (465/3238), and that with at least one segmental aneuploidy was 9.3% (302/3238); the percentage of blastocysts carrying mosaicism was 17.0% (549/3238). Eighty-nine blastocysts were tested unsuccessful (Fig. 1). Among the successfully analyzed TE biopsies, The percentage of blastocysts with euploidy, whole aneuploidy, segmental aneuploidy, and mosaicism was 63.8% (2008/3149), 14.8% (465/3149), 9.6% (302/3149), and 17.4% (549/3149) respectively.

### Main analysis

The number of euploid blastocysts, W-aneuploid blastocysts, S-aneuploid blastocysts, and mosaicism blastocysts are summarized in terms of biopsy day, degree of expansion, ICM grade, and TE grade in Table 2. Analysis by chi-squared test showed that the embryo development speed (biopsy day), expansion degree, and ICM grade were significantly associated with euploidy and W-aneuploidy but not with the incidence of S-aneuploidy or mosaicism in blastocysts. Among the blastocysts, the rates of euploidy were significantly higher, and the rates of W-aneuploidy were significantly lower on biopsy day 5, at expansion degree 6, and at a higher ICM grade. Among the TE grades (A, B, C), there were significant differences in euploidy, W-aneuploidy, S-aneuploidy, and mosaicism. TE grade C tended to result in a lower proportion of euploidy and higher proportions of W-aneuploidy, S-aneuploidy, and mosaicism (Table 2).

**Table 2:** Incidence of euploidy and chromosomal abnormalities at different TE biopsy days and blastocyst morphological parameters

Variables	Euploid blastocysts n (%)	*P* value	W-aneuploid blastocysts n (%)	*P* value	S-aneuploid blastocysts n (%)	*P* value	Mosaic blastocysts n (%)	*P* value
Biopsy day								
Day 5 (*n* = 684)	487 (71.2%)	< 0.001	62 (9.1%)	< 0.001	57 (8.3%)	0.380	107 (15.6%)	0.265
Day 6 (*n* = 2401)	1486 (61.9%)		390 (16.2%)		240 (10.0%)		428 (17.8%)	
Day 7 (*n* = 64)	35 (54.7%)		13 (20.3%)		5 (7.8%)		14 (21.9%)	
Expansion degree								
4 (*n* = 82)	43 (52.4%)	0.001	17 (20.7%)	0.011	9 (11.0%)	0.065	16 (19.5%)	0.718
5 (*n* = 2449)	1536 (62.7%)		378 (15.4%)		249 (10.2%)		431 (17.6%)	
6 (*n* = 618)	429 (69.4%)		70 (11.3%)		44 (7.1%)		102 (16.5%)	
ICM grade								
A (*n* = 437)	319 (73.0%)	< 0.001	36 (8.2%)	< 0.001	36 (8.2%)	0.571	65 (14.9%)	0.207
B (*n* = 2530)	1583 (62.6%)		390 (15.4%)		249 (9.8%)		447 (17.7%)	
C (*n* = 182)	106 (58.2%)		39 (21.4%)		17 (9.3%)		37 (20.3%)	
TE grade								
A (*n* = 414)	303 (73.2%)	< 0.001	37 (8.9%)	< 0.001	34 (8.2%)	0.024	56 (13.5%)	0.009
B (*n* = 1908)	1269 (66.5%)		242 (12.7%)		169 (8.9%)		325 (17.0%)	
C (*n* = 827)	436 (52.7%)		186 (22.5%)		99 (12.0%)		168 (20.3%)	

Values are presented as numbers (percentages), W-aneuploidy, whole chromosomal aneuploidy, S-aneuploidy, segmental chromosomal aneuploidy, ICM, inner cell mass, TE, trophectoderm

Logistic regression analysis was conducted to evaluate the contributions of female age, male age, biopsy day, degree of expansion, ICM grade, and TE grade as predictors of chromosome abnormalities. Male age had no significant correlation with euploidy or chromosomal abnormalities in blastocysts. The proportion of euploid blastocysts decreased and W-aneuploid blastocysts increased significantly with advancing maternal age (*P* < 0.001, OR 0.94, 95% CI 0.92–0.97; *P* < 0.001, OR 1.14, 95% CI 1.10–1.19, respectively). The proportion of euploid blastocysts was significantly lower among blastocysts biopsied on day 6 or 7 than among those biopsied on day 5 (*P* < 0.001, OR 0.69, 95% CI 0.56–0.84; *P* = 0.032, OR 0.55, 95% CI0.32–0.95, respectively). ICM grade C blastocysts had significantly lower euploidy (*P* = 0.016, OR 0.63, 95% CI 0.43–0.92) and significantly higher W-aneuploidy (*P* = 0.001, OR 2.37, 95% CI 1.41–4.01) than ICM grade A blastocysts. TE grade B and TE grade C blastocysts had significantly lower euploidy than TE grade A blastocysts (*P* = 0.038, OR 0.77, 95% CI 0.60–0.99; *P* < 0.001, OR 0.47, 95% CI 0.36–0.63, respectively), and TE grade C blastocysts had significantly higher W-aneuploidy than TE grade A blastocysts (*P* < 0.001, OR 2.42, 95% CI 1.60–3.64). However, female age, day of TE biopsy, degree of expansion, ICM grade, and TE grade were not associated with the proportions of S-aneuploidy and mosaicism in blastocysts, except that TE grade C blastocysts had significantly higher mosaicism than TE grade A blastocysts (*P* = 0.013, OR 1.57, 95% CI 1.10–2.23) (Table 3).

**Table 3:** Logistic regression analysis to evaluate the contributions of female age, male age, biopsy day, and morphological parameters as predictors of chromosomal abnormalities in biopsied blastocysts

Variables	Euploid blastocysts		W-aneuploid blastocysts		S-aneuploid blastocysts		Mosaic blastocysts	
	*P* value	OR (95%CI)	*P* value	OR (95%CI)	*P* value	OR (95%CI)	*P* value	OR (95%CI)
Female age	< 0.001	0.94 (0.92–0.97)	< 0.001	1.14 (1.10–1.19)	0.191	1.03 (0.98–1.09)	0.697	0.99 (0.96–1.03)
Male age	0.198	0.99 (0.96–1.01)	0.316	0.99 (0.96–1.05)	0.224	0.98 (0.94–1.02)	0.451	1.01 (0.98–1.04)
Biopsy day								
Day 5	0.001	Ref	0.001	Ref	0.308	Ref	0.431	Ref
Day 6	< 0.001	0.69 (0.56–0.84)	< 0.001	1.73 (1.28–2.34)	0.146	1.27 (0.92–1.74)	0.280	1.15 (0.90–1.47)
Day 7	0.032	0.55 (0.32–0.95)	0.058	1.97 (0.98–3.97)	0.960	0.98(0.37–2.57)	0.297	1.41 (0.74–2.68)
Expansion degree								
4	0.001	Ref	0.016	Ref	0.061	Ref	0.716	Ref
5	0.310	1.27 (0.80–1.99)	0.750	0.91 (0.52–1.61)	0.962	1.02 (0.50–2.07)	0.823	0.94 (0.54–1.64)
6	0.017	1.80 (1.11–2.92)	0.111	0.60 (0.33–1.12)	0.312	0.67 (0.31–1.45)	0.587	0.85 (0.47–1.54)
ICM grade								
A	0.051	Ref	0.005	Ref	0.940	Ref	0.543	Ref
B	0.100	0.81 (0.64–1.04)	0.073	1.43 (0.97–2.10)	0.759	1.06 (0.72–1.58)	0.763	1.05 (0.77–1.42)
C	0.016	0.63 (0.43–0.92)	0.001	2.37 (1.41–4.01)	0.756	1.11 (0.59–2.07)	0.294	1.28 (0.81–2.04)
TE grade								
A	< 0.001	Ref	< 0.001	Ref	0.129	Ref	0.038	Ref
B	0.038	0.77(0.60–0.99)	0.155	1.32 (0.90–1.95)	0.684	1.09 (0.73–1.63)	0.120	1.29 (0.94–1.78)
C	< 0.001	0.47 (0.36–0.63)	< 0.001	2.42 (1.60–3.64)	0.119	1.42 (0.91–2.21)	0.013	1.57 (1.10–2.23)

OR, odds ratio, CI, confidence interval, W-aneuploidy, whole chromosomal aneuploidy, S-aneuploidy, segmental chromosomal aneuploidy, ICM, inner cell mass, TE, trophectoderm

### Subanalysis

**Fig. 2:**
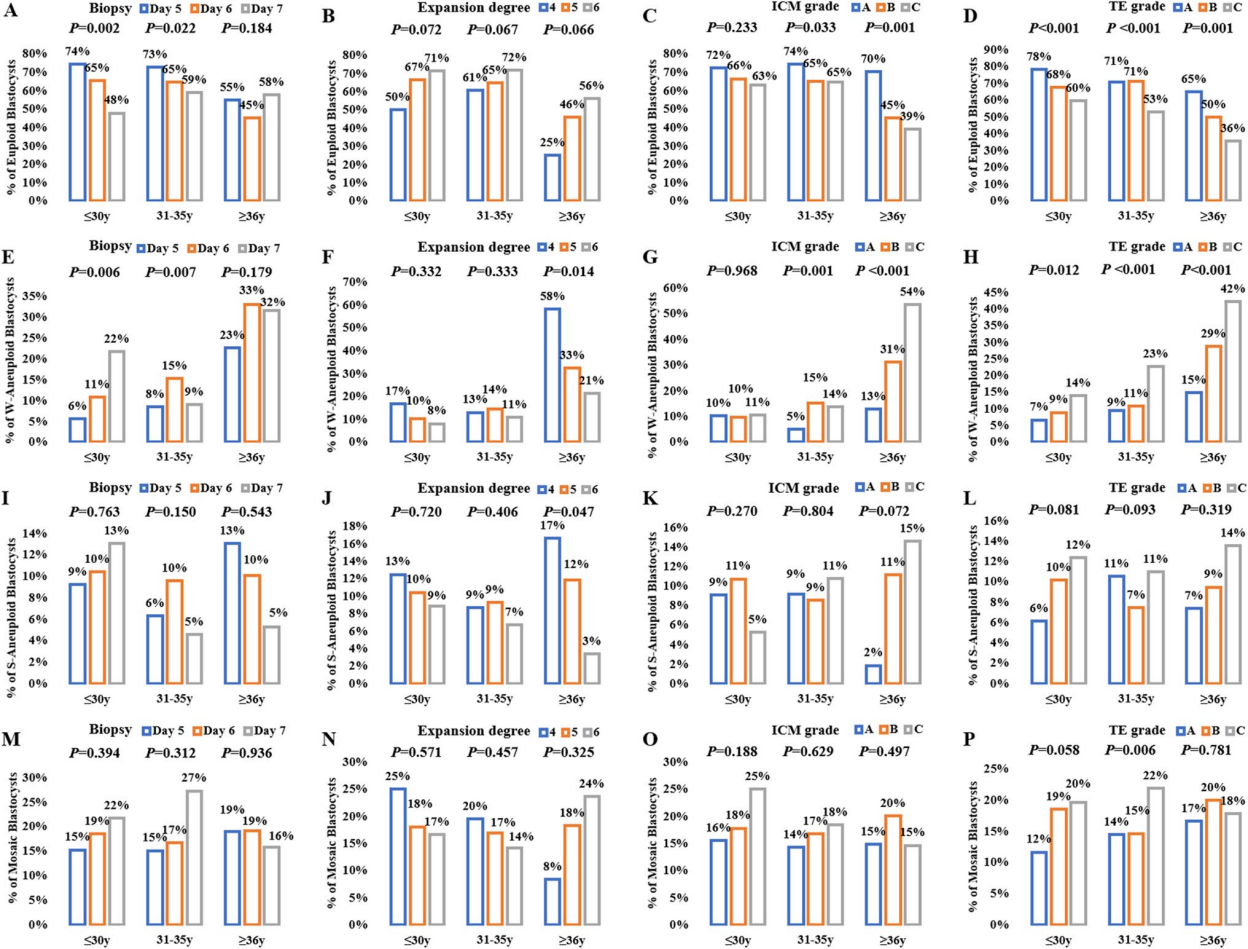
The chromosomal abnormalities at different biopsy days, expansion degrees, ICM grades, and TE grades for different female ages. (A ~ D) Incidence of euploidies (E ~ H) Incidence of W-aneuploidies. (I ~ L) Incidence of S-aneuploidies. (M ~ P) Incidence of mosaicism

For further subanalysis, data were divided into three groups according to female age (≤ 30 y, 31–35 y, ≥ 36 y), and the results are summarized in Fig. 2. The correlation of embryo development speed and morphologic parameters with euploidy and chromosomal abnormalities appeared to vary by female age. The day of TE biopsy had significant effects on euploidy and W-aneuploidy in the groups of women aged ≤ 30 y and 31–35 y, while expansion degree did not affect euploidy or W-aneuploidy in these age groups. In the group of women aged ≥ 36 y, W-aneuploidy increased significantly in blastocysts with expansion degree 4 compared to those with expansion degrees 5 and 6. The ICM grade was significantly positively correlated with euploidy and significantly negatively correlated with W-aneuploidy in the groups of women aged 31–35 y and ≥ 36 y but not in the group of women aged ≤ 30 y. TE grade was significantly positively correlated with euploidy and significantly negatively correlated with W-aneuploidy in all female age groups. However, S-aneuploidy and mosaicism had no correlation with the biopsy day, expansion degree, ICM grade, or TE grade in any age group, except that the expansion degree was significantly negatively correlated with S-aneuploidy in women aged ≥ 36 y (*P* = 0.047) and the mosaicism rate was significantly higher in TE grade C than in TE grades A and B among women aged 31–35 y (*P* = 0.006), though this may be due to the small sample sizes in some subgroups (Fig. 2).

## Discussion

In this study, we demonstrated that male age had no significant correlation with euploidy or chromosomal abnormalities in blastocysts. We found that female age, biopsy day, blastocoel expansion degree, ICM grade, and TE grade were associated with euploidy and W-aneuploidy, and these factors were not associated with S-aneuploidy or mosaicism except that TE grade seemingly has a weak correlation with segmental aneuploidy and mosaicism, in blastocysts detected by TE cells. The contribution of these factors to euploidy and W-aneuploidy varies by female age.

The subjects included in this study were patients who underwent treatment for PGT-M in a single reproductive center. This has several significant strengths. First, the therapeutic methods and techniques used in the clinic and laboratory are relatively unified. Second, compared to most previous studies[26–30], the subjects in this study had a low mean maternal age (31.8 ± 4.0 years) and a large age range (22–43 years), making them more representative of infertile people. Last, some factors for embryonic chromosomal abnormalities, such as parental chromosome structural rearrangement, recurrent miscarriage, and repeated IVF failure, were excluded. Thus, the data of this study can more directly reflect the relationship between blastocyst morphology and de novo chromosomal abnormalities.

Aneuploidy in embryos is the main reason for implantation failure and abortion in IVF cycles and increases with advancing maternal age[31, 32]. Our data confirm this finding: euploidy was significantly lower and W-aneuploidy was significantly higher among women of the advanced age than among young women, and logistic regression analysis showed that the aneuploidy rate increased by ~ 14% per year of female age, which is consistent with the previous studies[13, 33]. Franasiak et al. reported that the rate of aneuploidy in women aged ≤ 23 years is 40%, and that in women aged 26–30 years is the lowest, at approximately 20%-27%. The incidence of aneuploidy increases gradually from 31 to 43 years of age, reaching a high plateau of approximately 85%[13]. Study by Nair et al. showed the incidence of aneuploidy rate was ~ 28% at maternal age < 30 years which steadily increased to ~ 67% in women above 40 years [33]. However, male age had no effect on the euploidy and chromosome abnormalities of embryos. In laboratories, morphological evaluation has been the main strategy applied to choose the embryos to transfer. Most reports demonstrate that the implantation, clinical pregnancy and live birth rates in the day-5 blastocyst group are significantly higher than those in the day-6 blastocyst group[12, 26, 34] and that morphologic parameters (degree of expansion, ICM grade, and TE grade) are significantly positively correlated with the outcomes after blastocyst transfer[27, 29]. Some studies suggested that the transfer of euploid blastocysts tested by PGT-A can lead to higher rates of clinical pregnancy, live birth, and implantation than those of non-PGT-A tested blastocysts [35, 36]. However, some studies showed that PGT-A could not enhance the ongoing pregnancy rate[37] and live birth rate[38] in young patients, which may be due to the fact that embryos from young patients have low aneuploidy. In regard to chromosomes, the rate of euploidy is significantly higher among blastocysts biopsied on day 5 than among those biopsied on day 6 or day 7[39]. Aneuploid blastocysts have significantly delayed development compared with euploid blastocysts at the start of compaction[40]. The average euploid blastocyst expansion rate is significantly faster than that of aneuploid blastocysts. Some studies suggest that a top-quality ICM and TE, higher expansion grade, and shorter time to the start of blastulation have a higher percentage of euploid blastocysts[30, 39] and a lower incidence of de novo chromosomal abnormalities[41]. The conclusions about the relationship of morphological parameters with euploidy and aneuploidy are consistent with its effect on clinical outcomes, and our results are in accordance with these previous studies at the blastocyst chromosomal level. This further suggests that the combination of female age, embryo developmental speed, and the three morphologic parameters can be used to build algorithms to predict embryonic ploidy and clinical success in ART treatment.

Most studies on embryo aneuploidy often refer to whole chromosomal aneuploidy. However, few studies have focused on the relationship between the incidence of segmental aneuploidy and embryo morphological parameters. A dataset from Escribà et al. showed that there was no relationship between the incidence of segmental aneuploidy and the day of blastocyst biopsy or blastocyst stage, but the rate of segmental aneuploidy was significantly higher among blastocysts with poor ICM and TE quality[42]. Liu et al. analyzed the incidence of de novo chromosome abnormalities related to embryo parameters in the cycles performed for chromosome structural rearrangement and PGT for aneuploidy[41]. The results showed that the incidence of de novo segmental chromosome abnormalities was significantly related to TE grade but not to ICM grade or blastocyst development speed. In our study, the rates of S-aneuploidy increased with TE grade in the groups of women aged ≤ 30 y and ≥ 36 y, although the difference was not significant. The inconsistent results may be due to the different subjects and sample sizes included in the study. The average patient age in our study was younger than that of the abovementioned two studies[41, 42]. The proportion of S-aneuploid blastocysts was lower (9.6%) than that of euploid (63.8%) and W-aneuploid (14.8%) blastocysts. Most of the segmental changes in human blastocysts arise from mitotic errors[17, 43] and affect paternally derived chromosomes, which are probably due to sperm DNA fragmentation during spermatogenesis[19]. There are few studies on the relationship between sperm quality and the development and morphological parameters of blastocysts. Sperm quality, including sperm DNA fragmentation, has a significant correlation with male age[44, 45]. However, paternal age has no impact on fertilization rate, embryo quality, pregnancy, live-birth rates, or miscarriage rate[46, 47] and is not associated with rates of aneuploidy in embryos[19, 40]. The incidence of segmental aneuploidies was not related to female age[43], and sperm DNA fragmentation did not correlate with blastocyst aneuploidy or morphological grading[48, 49]. Therefore, we speculate that male factors have little influence on embryo morphology, which may be affected more by female age and egg quality, so the relationship between S-aneuploidy (mainly paternal origin) and embryo morphology is weak.

Mosaicism has been reported in cleavage- and blastocyst-stage embryo biopsies, and the incidence of mosaicism can vary greatly across laboratories due to differences in NGS protocols and reporting practices[40, 50–53]. In our study, the mosaicism rate among blastocysts was 17.4%. Mosaic embryo transfer could result in viable and genetically normal live births, although it has a significantly lower clinical pregnancy rate and ongoing/live birth rate and a significantly higher miscarriage rate than euploid and non-PGT transfers[54, 55]. A retrospective cohort study by Martín et al. compared the morphokinetic characteristics of 1,511 blastocysts classified as euploid, aneuploid, low-degree mosaic (30%-50%), and high-degree mosaic (50%-70%), and the results showed that embryo morphokinetics were not correlated with the degree of mosaicism or a mosaicism configuration.[40]. Another retrospective cohort study by Chen et al. used multivariate generalized estimating equation analysis to evaluate the correlation of time-lapse-based variables and numeric blastocyst morphological scores with different mosaic levels (≤ 20%, ≤ 50% and ≤ 80%) in 918 biopsied blastocysts and suggested that blastocyst morphokinetic are significantly correlated with the threshold levels of mosaicism[28]. The correlation between blastocyst morphology and the mosaicism level is unclear. In our study, we did not classify blastocysts based on the degree of chromosomal mosaicism. Our results suggested that there was no significant correlation between blastocyst morphology and the incidence of mosaicism, except that TE grade had a significant negative correlation with mosaicism in women aged ≤ 35 years. The reason for the inconsistency is probably that some of the mosaic embryos are false-positive diagnoses or that the mosaicism is the byproduct of a misdiagnosis by PGT-A[40]. Further studies are needed to increase the understanding of embryonic mosaicism and its impact on preimplantation dynamics.

It has been demonstrated that TE karyotype can be an excellent predictor of ICM karyotype[56]. Baatarsuren et al. suggested that the TE could be a better predictive parameter than ICM for live birth rate[26]. In our study, TE grade was closely related to euploidy and W-aneuploidy in all female age groups, while the day of biopsy was related to euploidy and W-aneuploidy in the women aged ≤ 35 years. The expansion degree was related to euploidy and W-aneuploidy in only the group of women aged ≥ 35 years, and ICM grade was related to euploidy and W-aneuploidy in the groups of women aged 31–35 years and ≥ 35 years. The effect of development speed, degree of expansion, and ICM grade on euploidy and W-aneuploidy was variable among the different female age groups, probably because female age is the main factor affecting egg quality, which leads to the differences in developmental potential among embryos. Additionally, TE grade had a weak negative relationship with S-aneuploidy and mosaicism in blastocysts, although most of the differences were not significant. The cells used to detect euploidy by NGS are from the TE biopsy, which is why TE morphology is more closely related to the NGS results. TE aneuploidy is an excellent predictor of ICM aneuploidy, as the majority of ICM results were concordant with the TE biopsies[57, 58]. Our study supports the conclusion that TE morphology is a better predictive parameter for embryo aneuploidy.

## Conclusion

In conclusion, our study suggests that female age, biopsy day, and blastocyst morphology (expansion degree, ICM grade, TE grade) are fundamental parameters to predict euploidy and whole chromosome aneuploidy in patients undergoing ART. Blastocysts from younger women biopsied at day 5 and having a higher expansion degree, ICM grade, and TE grade are most likely to be euploid. The predictive roles of these parameters, such as biopsy day, expansion degree, ICM grade, and TE grade, vary among different female age ranges. However, parental age, biopsy day, expansion degree, and ICM grade do not correlate with segmental aneuploidy or mosaicism, and TE grade has a weak correlation with segmental aneuploidy and mosaicism in embryos.

There are some limitations in this study. First, this is a retrospective study that has intrinsic limitations, and data on clinical outcomes after blastocyst transfer are lacking. Second, all the blastocysts included in this study can be used for biopsy for chromosome detection on days 5, 6, or 7, so this study did not include blastocysts with expansion degrees of 1, 2, and 3. Third, uniform single or multiple aneuploidies and the level of mosaicism in noneuploid blastocysts were not distinguished.

## Data Availability

Data will be made available upon request to the corresponding author.
